# The Use of Edge-Betweenness Clustering to Investigate Biological Function in Protein Interaction Networks

**DOI:** 10.1186/1471-2105-6-39

**Published:** 2005-03-01

**Authors:** Ruth Dunn, Frank Dudbridge, Christopher M Sanderson

**Affiliations:** 1 The Wellcome Trust Sanger Institute, Wellcome Trust Genome Campus, Hinxton, Cambridge CB10 1SA, UK; 2 MRC Biostatistics Unit, Institute of Public Health, Robinson Way, Cambridge CB2 2SR, UK; 3 MRC Rosalind Franklin Centre for Genomics Research, Hinxton, Cambridge CB10 1SB, UK

## Abstract

**Background:**

This paper describes an automated method for finding clusters of interconnected proteins in protein interaction networks and retrieving protein annotations associated with these clusters.

**Results:**

Protein interaction graphs were separated into subgraphs of interconnected proteins, using the JUNG implementation of Girvan and Newman's Edge-Betweenness algorithm. Functions were sought for these subgraphs by detecting significant correlations with the distribution of Gene Ontology terms which had been used to annotate the proteins within each cluster. The method was implemented using freely available software (JUNG and the R statistical package). Protein clusters with significant correlations to functional annotations could be identified and included groups of proteins know to cooperate in cell metabolism. The method appears to be resilient against the presence of false positive interactions.

**Conclusion:**

This method provides a useful tool for rapid screening of small to medium size protein interaction datasets.

## Background

Protein interaction datasets are typically presented as graphs (or networks), in which the nodes are proteins and the edges represent the interactions between the proteins. These graphs can be used to investigate the functions of unannotated proteins through their interactions with neighbouring annotated proteins. Protein interaction datasets frequently contain many false positives and false negatives, (Bader et al [1], von Mering et al [2]) but studies have shown that true positives are frequently associated with areas where there are many interactions between neighbours (clusters). For example Giot et al [3] used independent datasets to remove false positives from a large-scale protein interaction dataset and as a result were able to demonstrate that true positives had a strong positive correlation with the clusters. Spirin and Mirney [4] found that clusters of highly interconnected proteins are significant features of protein interaction networks. These could not have occurred by chance and are therefore likely to represent groups of proteins that have co-evolved to serve a common biological function. Identification of clusters is therefore likely to capture the biologically meaningful interactions in large scale datasets.

Edge-Betweenness clustering [5], the method used here, has been exploited in the social and ecological sciences to study communities [6] and in the study of biochemical pathways [7]. It has proved to be a useful and adaptable method. As discussed by Holme et al [7] edge-betweenness uses properties calculated from the whole graph, allowing information from non-local features to be used in the clustering. Many other clustering methods, which have proved useful for clustering protein interaction graphs, are based on calculation of local quantities such as node degree (number of attached edges) [8,9]. These 'local' methods will exclude nodes with a low degree e.g. the many prey nodes attached to their bait by a single edge, which are common in yeast two-hybrid (Y2H) datasets. Methods using whole graph properties will automatically include these poorly connected nodes in clusters [5], whilst a 'local' method would need to restore such nodes in a post-processing step [9]. Clusters created using edge-betweenness clustering are therefore useful when the information associated with these nodes is required. Other methods based on whole graph properties will also have this advantage, for example Markov Clustering [10]. A discussion of different clustering methods can be found in [11]

We applied the edge-betweenness method to a set of human protein interactions from our laboratory [12,13]. In these experiments interactions were identified using the Y2H method. For comparison, two datasets of yeast protein interactions [14,15] were also analysed. One yeast dataset also used the Y2H method [14] whereas the other was prepared using affinity purification [15]. The functions identified for clusters by the automatic method were compared with the expert biologists' interpretations presented in these papers.

## Results

### Allocation of GO terms

#### Differences in clustering between the datasets

The three datasets used differ in content, purpose, size, structure and species. A more detailed description of each dataset is given in the 'Methods' section and in Table 1, but briefly, the Gavin and Uetz datasets were large scale screens of the yeast proteome, not focused on particular metabolic pathways, whereas the Lehner dataset is focused on a few metabolic areas/complexes related to the human MHC class III region. While Lehner and Uetz both used the Y2H method to detect protein-protein interactions, Gavin used a combination of affinity purification and mass-spectroscopy. The two yeast datasets (Gavin and Uetz) have approximately 5× more nodes than the Lehner dataset. Whilst the Gavin and Uetz datasets have roughly the same number of nodes, the Gavin (affinity purification) dataset has twice as many edges (3145 vs 1498) as the Uetz (Y2H) dataset. The affinity purification method (Gavin) retrieves fairly stable complexes of proteins whereas the Y2H method detects direct protein-protein interactions which may be weak or transient.

From Tables 2 and 3 it can be seen that the affinity purification dataset gives much bigger clusters with the removal of a similar proportion of edges, when compared to the Y2H datasets. When 15% of edges were removed from the Gavin dataset, the clusters (with more than one member) had an average of 23 nodes whilst for Uetz the average was just over 7 nodes. The Lehner dataset fell between these values. Diagrams showing the Lehner dataset before and after clustering are presented in Additional files 11 and 12.

The choice of the number of edges removed needs to be guided by the dataset and problem under consideration. A number of criterion could be used. (i) Range of cluster sizes: To decide what a sensible distribution of cluster sizes would be, the range of sizes of clusters found by affinity purification was used as a guide. Gavin [15] reported the distribution of cluster sizes as follows:-51% had 1–5 nodes, 18% 6–10 nodes, 15% 11–20 nodes, 6% 21–30 nodes, 4% 31–40 nodes, and 6% > 40 nodes. In order to emulate this type of distribution with the automatic clustering (see Table 2) it is necessary to remove more than 13% of edges from the Uetz and Lehner datasets and more than 25% from the Gavin dataset. Therefore it is necessary to remove a much higher proportion of edges from the affinity purification dataset.

Other results from Tables 2, 3 and 4 that could also be used to try and determine the appropriate number of edges to remove are (ii) increasing the significant number of GO terms per protein (iii) aiming for an average size of cluster of 5–20 proteins (iv) reducing the size of the biggest cluster to < 20% of the dataset, a useful metric to indicate reasonable decomposition of the dataset (but which could be varied according to the total number of nodes in the dataset) (v) reducing the number of nodes not associated with any other nodes to < 30%. The proportion of edges that need to be removed in order to attain each of these criteria would be:-

(i)distribution cluster size Gavin 25% Uetz 13% Lehner 14% edges

(ii) significant GO terms For all datasets, the more edges that are removed the more terms become significant down to the smallest cluster sizes investigated

(iii)average cluster 5–20 Gavin 25% Uetz 2–13% Lehner 7–25% edges

(iv)biggest cluster < 20% Gavin 25% Uetz 13% Lehner 14% edges

(v)single nodes < 10 % Gavin 25% Uetz 27% Lehner 25% edges

The data above shows that most of these criteria give similar results and suggest that the method used to produce the data (Y2H or affinity purification) will be a major determinant of the proportion of edges to remove. To summarise, for Y2H, useful results are obtained by removing 10%–15% of edges whereas for affinity purification, removing 25% edges gives better results. Newman and Girvan [16] have developed methods for assessing the 'modularity' of the clusters produced by edge-betweenness clustering. It would also be possible to use methods of this type, as a more objective way of deciding how many edges to remove in different datasets.

Size of cluster is important, because the quantity of significant annotation information i.e. the average number of significant GO terms per protein, (Table 4) increased, for all datasets, as cluster size decreased. However the detail of the information, measured as average depth of GO per node, did not change with cluster size. It is noticeable that human proteins in the Lehner dataset [12,13] had been annotated to a greater level of detail (average depth of nearly 6 in the GO hierarchy) than the yeast proteins (average dept of approx 4.7, see Table 4) and whereas virtually all of the clusters in the Lehner dataset had a correlation with at least one GO term there were many clusters in the yeast dataset which had no significant GO terms (the majority in the case of the Uetz dataset). This could be a peculiarity of the metabolic areas chosen for the Lehner study.

#### Scaling

The utility of this approach is currently restricted by the size of the dataset being analysed, especially when a large number of edges are being removed. For the Gavin dataset, when 57 edges were removed the total time to cluster was 1 h 25 min but when removing 1500 edges it took 10 h 10 min. According to the software documentation [17] and as discussed by Newman [6] the running time for sparse graphs (such as these) is proportional to both the number of edges removed and the total number of nodes. The Ito dataset (see below) took >>24 h when > 500 edges were removed. This method is therefore of greater utility for small to medium datasets, having less than 2000 nodes or edges.

#### Significance of GO terms

After performing the Chi Squared tests and checking them against a random reallocation of GO terms across the network, all the significant GO cluster correlations remained significant. In no case were more than 5% of the lowest p values of the randomly reallocated GO terms lower than the lowest p value in the original dataset.

In almost every case the significant annotations were informative about a potential function for the clusters (see Table 5), providing distinctive groupings of annotations which distinguished different functions for the different clusters (the aim of the method). It was often a very small proportion of the proteins which provided the annotations which were used to characterise the cluster, (Table 5 and Additional Files 1, 2, 3, 4, 5, 6, 7, 8, 9, 10, which provide complete sets of clustering results and details of the proteins which contributed the significant annotation).

### Correlation with biological function

One test of this method was to determine whether the clusters generated and the associated GO terms corresponded to clusters previously identified by expert biologists.

With respect to the Lehner et al dataset [12], the authors identified groups of interacting proteins which appeared to be involved in distinct biological processes including transcription regulation, protein-ubiquination, cell cycle regulation and mRNA processing

When edge-betweenness clustering was used to remove 57 edges, 21 clusters (with size greater than one) were created (Table 3). From Table 5 (and from the more detailed information in Additional file 7), it can be seen that these clusters differ in the significant GO terms associated with them i.e. the method does separate groups of proteins with different metabolic functions. Significantly, clusters were generated with functions corresponding to all of the metabolic areas identified by informed biological interpretation. These were transcription (cluster 9), ubiquination (cluster 15), cell cycle reg (cluster 14) and mRNA processing (cluster 21, cluster 3, cluster 6) Only one cluster, cluster 10, had a description ("biological process") which was too general to give useful information about function. However when this cluster was broken down further (in the test with 100 edges removed) more informative terms ("response to abiotic stimulus", "eukaryotic translation elongation factor 1 complex") were associated with the new, smaller parts of the cluster. Interestingly cluster 10 contained very few proteins with GO terms assigned to them and therefore may represent an under-investigated module in the human proteome. This highlights the dependence of this method on the quality (depth) and quantity of the GO annotations available. This was good for the *H.sapiens *proteins but less good for the yeast proteins.

One important question is whether the functions identified for these protein clusters are confirmed by biological experimentation. The Lsm complex is mentioned by the authors of all 3 papers [13-15], It has been extensively studied in both yeast and human [13]. The Lsm complex has been shown to have a number of functions related to RNA processing, including the splicing of nuclear pre-mRNA and the decapping of cytoplasmic mRNA prior to degradation.

#### Clusters in the Lehner dataset

In the Lehner dataset two GO terms, GO:6371 "mRNA splicing" and GO:8380 "RNA splicing", were always associated with only one cluster in the dataset. This was a good candidate for the Lsm complex.

Of the 8 Lsm proteins examined in [13], all eight were found in the cluster associated with these two GO terms for the tests when 10, 30 and 57 edges were removed. A diagram showing the cluster containing these proteins, in the dataset with 57 edges removed, can be seen in Additional file 13. When 100 edges were removed, the cluster labeled as RNA splicing contained 5/8 of the Lsm proteins. The three clusters containing the other 3 proteins had the following significant descriptions (the number in parenthesis shows; the number of proteins with this annotation / the total number of proteins in the cluster).

GO:15980 energy derivation by oxidation of organic compounds (2/19)

GO:5837 26S proteosome (2/16)

GO:6350 transcription (2/3)

For the Lehner data, when 15, 30 and 57 edges were removed, the clusters labeled as being associated with RNA splicing are large containing 190, 143 and 60 proteins respectively (see below). The cluster with 5/8 Lsm proteins (100 edges removed) had only 17 proteins. In addition to the Lsm proteins the large clusters contained other proteins known (i.e. having GO labels) to be involved in RNA splicing. The proportions are shown in Table 13.

This data clearly shows that as the cluster size gets smaller, the cluster is more focused round the RNA splicing function. Larger clusters must have sub-clusters related to other functions. The last column in the table above shows that many of the RNA splicing proteins grouped in these clusters were the prey of the Lsm proteins in the original experiments [13], which is what we hoped this method would achieve.

Therefore for the Lehner data, the cluster identified by Edge-Betweenness clustering as the "RNA splicing" cluster, did contain the proteins expected to be associated with this process. However this is a small dataset focused around a specific biological process. A more stringent test of this method is provided by the yeast proteome datasets where screening was not functionally focused.

#### Clusters in the yeast datasets

Gavin et al [15] and Uetz et al [14] both describe the Lsm complex. One complication in both of these datasets, is that the yeast proteins are not annotated to the same level of detail as the human proteins. For example there is no annotation for "RNA splicing" but only the higher level GO term GO:16070 "RNA metabolism", which covers a much broader range of cellular processes.

In Gavin et al [15], the Lsm proteins are found in the complex described as TAP-C128. This contained 36 proteins. The distribution of the TAP-C128 proteins between the clusters are shown in Table 6. It can be seen that a minimum of 6/7 Lsm proteins and proteins associated with RNA metabolism are clustered together, at all numbers of edges removed.

Therefore in a dataset not focused round RNA metabolism, the edge-betweenness algorithm successfully clustered the Lsm proteins with a number of other proteins that were co-purified in the TAP-C128 complex and a cluster produced using the graph topology was shown to correspond to a cluster of known function.

In Uetz et al 2000 [14], the Lsm complex is described as a set of 16 interacting proteins. The one cluster containing all of these proteins does not correlate with the GO term for "RNA metabolism" in the datasets with 30 or 57 edges removed. This correlation only emerged once 100 edges had been removed. With 400 edges removed 11/16 are still in the same "RNA metabolism" cluster (the other 5 are spread between 5 different clusters).

Therefore in the Uetz dataset although all the Lsm proteins clustered together, it was only once more than 10% of edges had been removed that it was possible to get a significant association with the relevant GO term. Finding the correct number of edges to remove is obviously essential to extracting the required information.

Overall it can be seen that the method is capable of finding clusters of proteins with known biological function and of correctly assigning a relevant annotation to a particular group.

#### Stable and transient clusters

In Gavin et al [15] the authors discuss two clusters which are described as "stable and "transient". TAP-C162 is an example of a "stable" complex which was always isolated with the same members. It is part of the poly-adenylation machinery. In contrast, TAP-C151, the "transient" complex was frequently isolated with different components. It is a signaling complex formed around protein phosphatase 2a.

The distribution of these two complexes between the clusters generated by edge-betweenness clustering, was compared at different levels of clustering, (see Tables 7 and 8). While TAP-C162 remains mainly associated with one cluster at all numbers of edges removed, TAP-C151 becomes distributed much more evenly between a greater number of clusters. Therefore it seems likely that the method described here favours the detection of more stable clusters, as the number of edges removed increases.

### False positive interactions

Clustering the Lehner dataset with added false positive edges (see "Methods" section and Table 9) gave no obvious difference in cluster size (Tables 10 and 11) or quality or quantity of GO annotation (Table 12). The dataset with false positives is slightly larger than the original dataset, but this did not change the number of clusters. The slight increase in average cluster size led to a commensurately small fall in annotation quality (GO per node), but there were no dramatic differences in cluster size distribution or any of the other measurements.

Fourteen out of twenty-one of the clusters in the original dataset remained completely intact, and even when this was not the case a minimum of 70% of the original proteins in the other clusters could still be found together in one of the new clusters. Therefore adding the false positives did not render any of the original clusters unrecognisable.

When the dataset with the false positive edges removed was compared to the dataset with the same number of edges removed at random, the differences were more marked. The dataset where edges were removed at random had smaller clusters (Tables 10 and 11) and more single nodes (Table 11 last column). The identity of the clusters was perturbed to a greater extent. Further analysis showed that when the false positives were removed 12/21 clusters still remained completely intact. With removal of random edges only 4/21 clusters were completely intact. However even in this dataset 14/21 clusters had 80% of proteins from the original clusters co-occurring i.e. 3/4 of clusters were still recognisable. Randomly removed edges can be considered to be false negatives and so the method is also showing good tolerance to false negatives, and can still preserve a good level of cluster identity.

Overall, even though the false negatives reduce the average sizes of the clusters and splits off many single nodes (as would be expected because nodes with single edges are much more abundant than nodes with multiple edges, in Y2H datasets) the same clusters are still being found 75% of the time. In other words the presence of false positives and false negatives in the dataset does not seem to distort the composition of the clusters created by the Edge-Betweenness method in a way that obliterates cluster identity. But false negatives do appear to have a slightly more detrimental effect than false positives.

Looking at the edges which were removed during clustering, when 57 edges were removed (from the dataset containing false positive edges) 3/57 (5%) had false positive nodes at one or both ends. When clustering was done by removing 100 edges 15/100(15%) were attached to false positive nodes. This compares with 68/465(14.6%) edges attached to false positive nodes in the whole dataset. There is no obvious bias in the presence of false positive edges between or within clusters.

Overall it appears that the clustering is fairly robust to the presence of false positives and also to the random removal of edges i.e. false negatives.

With the Ito et al [18] dataset it was hard to say whether there was much effect from the removal of false positives or addition of false negatives, as the proportion of nodes and edges affected was so small, but again there were no obvious differences.

## Discussion

Edge-Betweenness clustering can be used to separate protein interaction networks into clusters which have correlations with annotated gene functions. This can be done in an automated fashion and thus can provide a means of rapidly screening the results of protein interaction experiments. Clusters produced by this method contain groups of proteins which are known to cooperate to perform common functions, described by the correlating annotations. Therefore the clusters detected by this method correspond to active protein complexes found in the cell. Moreover the method worked for different types of dataset (Y2H and affinity purification) different organisms (yeast and human) and for datasets with a 5× difference in the number of edges.

The smaller the clusters generated by this method, the higher the average number of significant annotations. The preliminary results presented here suggest that, in general, useful information was obtained once approximately 10% of edges were removed from Y2H datasets and a slightly higher proportion (25%) from affinity purification data. This method is particularly good at detecting "stable" clusters. The method is also flexible and can be adjusted according to the nature of the dataset and to the function being studied. Currently scaling to very large datasets when large numbers of edges need to be removed is problematic, but this may soon be alleviated by new developments of the algorithm [6]. The level of detail and amount of available annotation will have a significant effect on the utility of this method although it is possible to tune the amount of annotation found by the method, by altering the number of edges removed. The amount of available annotation will increase as proteome annotation progresses.

Spirin and Mirny [4] have demonstrated the robustness to false positives and negatives of various clustering methods (not including the Edge-Betweenness method used here). They found that 80% of clusters could still be detected if up to 20% of links were added or removed. Our results suggest that Edge-Betweenness clustering is similarly robust. This robustness is undoubtedly for the reason identified in [4] which is "the use of multiple interactions to identify a cluster", in other words the interconnectedness of a pair of proteins is reconfirmed by the interconnectedness of their neighbours. The biological significance of these interconnected sets of proteins was shown by the high correlation between true positive interactions and clusters in *Drosophila *protein interaction networks, found by Giot et al [3].

Giot et al [3] also found that prey (but not bait) with a large number of neighbours had a significant negative correlation with the reliability of the interactions. These highly connected prey correspond to the promiscuous prey which we identified as false positives and which although highly connected do not have neighbours which are themselves highly interconnected. As this method appears robust to the presence of such proteins it is not necessary to "clean up" the datasets before using them.

The hierarchical nature of the Gene Ontology made this a very useful system of annotation to exploit in this method. It allows proteins to be grouped according to the most detailed shared level of annotation but also enables higher level (less informative) annotation to be used when this is all that is available. The very high level terms which apply to almost all proteins are usually ignored as they are not concentrated in a particular cluster, although these terms occasionally appear as significant, in clusters with higher than average levels of annotation.

## Conclusion

Edge-Betweenness clustering provides a quick way of picking out functionally interesting areas of protein interaction datasets. It also appears to be robust against false positives and negatives. As such this approach can be applied to any quality of data. It also deals effectively with poorly connected nodes, such as the many prey with single connections found in Y2H graphs. Because the Edge-Betweenness algorithm does not scale well to larger graphs, this method is currently most appropriate for studies focused on specific areas of the proteome. However, modifications of the algorithm are being developed and these should allow it to be applied to larger datasets in the future [6]. The implementation described here is particularly effective where good quality GO annotation is available, which is especially true for many human proteins. It will be a useful method for detecting functions for unannotated proteins based on the knowledge of the functions of their neighbours and for exploring functional modules within the proteome.

## Methods

### Datasets

The datasets used for analysis are described in Table 1. Briefly the Lehner dataset comes from our work on the function of the MHC class III region [12,13] and is a small, highly focused dataset of *H. sapiens *protein interactions, detected using the Y2H method [12]. The other datasets, Gavin [15] and Uetz [14], are larger datasets resulting from mass screens of the yeast proteome, using either Y2H (Uetz) or affinity purification (Gavin). The method presented here was developed for the Lehner dataset. In order to test the method, it was applied to the larger, less selective yeast datasets.

The Ito dataset [18], an even larger yeast dataset, was included in order to test the effect of false positive proteins. This dataset contained 16 proteins identified by Gavin et al [15] as false positives. However it was not used for other aspects of the investigation as clustering takes a long time when large number of edges are removed. Thus the Ito dataset represents the upper limit of the size of datasets suitable for use with the method described here.

### Protein function

The Gene Ontology (GO) [19] was used as the source of functional annotations. It was chosen because it provides hierarchically structured, controlled vocabularies. Genes or gene products may be labeled with terms from any level in any of the three hierarchies (ontologies). By searching up through the hierarchy, it was possible to find terms shared by proteins which had been initially labeled with different descriptions. The search through the hierarchy is easy to automate, which makes it possible to group together proteins participating in the same general functions, even when they were originally annotated for different, more specific functions.

### Steps of the analysis

The steps of our method to cluster the graph and assign functions to the clusters, were as follows:-

1. Transform the protein interaction data to GraphML (an XML format for graphs [20]), removing any parallel edges, to make the data ready for import into JUNG.

2. Use the JUNG graph analysis framework [21] to cluster the data using the "Edge-Betweenness" [5] algorithm.

3. Find GO terms and the parents of those GO terms for each GO annotated protein in every cluster.

4. Test the association between each GO term and each cluster, from a 2 by 2 contingency table.

5. Correct the association tests for multiple comparisons, using a permutation test with random re-allocation of GO terms to proteins.

6. Generate reports on cluster size and significant GO terms.

Perl scripts were used to perform most of these steps, the other software used is described below. Details of the steps listed above are as follows:

### Clustering

JUNG version 1.3 [21] was used to cluster the graph by the Edge-Betweenness clustering method [5]. This algorithm removed those edges which lay on routes between interconnected clusters. "Betweenness" is calculated by finding the shortest path(s) between a pair of vertexes and scoring each of the edges on this/these path(s) with the inverse value of the number of shortest paths. (So if there was only one path of the shortest length, each edge on it would score 1 and if there were 10 paths of that length, each edge would score 1/10.) This is done for every pair of vertexes. In this way each edge accumulates a "betweenness" score for the whole network. The network is separated into clusters by removing the edge with the highest "betweenness", then recalculating betweenness and repeating until the desired number of edges have been removed. The method is fully described in [5].

The number of edges to remove was supplied as a parameter. Removing a larger number of edges reduced the size of the clusters produced. The number of edges removed was varied to see whether (a), clusters of certain sizes gave better correlations with GO terms and (b), whether datasets of different types cluster in different ways (likely, as the affinity purification dataset has approximately 3× as many edges as the Y2H dataset with a similar number of nodes).

### Source of GO annotations

GO terms available for each of the proteins in the graph were retrieved. In the case of the Lehner dataset these were taken from the RefSeq records [22], for the Uetz and Gavin data these were provided by BIND [23].

### Processing GO annotations

The Gene Ontology "termdb" release from December 2003 was used as the source of the parent GO terms [24]. Tables to hold these GO data were set up using the PostgreSQL relational database management system [25] (version 7.3.4-RH). The parents of each GO term were found by using an adaptation of the sample query provided on the GO web site [26]. This query was called from either perl scripts or Java programs, which allocated the terms to the clusters.

### Detecting GO terms with significant associations to clusters

The 'R' statistical package [27] (version R 1.8.1 (2003-11-21)) was used to perform the statistical analysis on the data retrieved. The association between each cluster and each GO term was tested using a 2 by 2 contingency table by Fisher's exact test.

### Re-testing significant GO associations

The GO terms (significant and non-significant) were redistributed across the clustered network at random. The p value was recalculated for each GO/cluster combination. This randomisation was repeated 1000 times. The overall significance was calculated as the proportion of randomisations in which the smallest p value for a GO-cluster association was less than or equal to the smallest p value in the original data. We considered the GO numbers to be significantly associated with the clusters if the overall significance was less than 5% (i.e. fewer than 50 of the 1000 randomisations' lowest p values were smaller than the smallest p value from the observed data).

### Reports on significant GO/cluster associations

In order to compare the informativeness of the GO/cluster associations, the following ratios were calculated (a), the average number of GO terms per node in the clusters and (b), the average depth of the GO terms per node per cluster. These provided an indication of the 'quantity' and 'quality' of the GO information. A GO at a greater depth in the GO hierarchy provides more detailed information than one higher in the GO hierarchy.

### False positives

In our original experiments [12,13] there were a number of prey that interacted with many different bait. Prey found by more than three different bait were defined as false positives (of the 'promiscuous' type). There were 14 of these (approximately 4% of the dataset nodes). 10 of these 14 had been excluded from the original data. To investigate their effect on clustering, these nodes and all associated edges were added back to the data. This contributed 59 new edges to the dataset (13% of dataset edges). This dataset was clustered and the clusters compared to those found in the original experiment.

If these nodes were disconnected this removed 68 edges, so nine of the edges connected to false positives were part of the original data. In a control experiment 68 edges were removed at random (from the dataset with false positives added), this dataset was clustered. This was repeated 100 times and the results were compared to the clusters obtained from the dataset which had false positive edges removed.

Gavin 2002 Supplementary Information Table S2 [15] provided a list of false positive proteins, which were excluded from their yeast dataset. They were excluded because they either appeared in more than 20 of the purifications or were isolated in mock transformations. The data describing the edges created by these proteins was not provided, therefore it was not possible to add them back to the Gavin data. The Uetz data contained only 2 of the false positive proteins, however the Ito dataset contained 16(0.5% of dataset). The Ito dataset is large and 16 out of 3271 nodes is a very small proportion, so any effect will not be large. Disconnecting these nodes removed 26 edges from the dataset (0.6% of edges). A control dataset had 26 edges removed at random before clustering

All false positive datasets (see Table 9) and controls were clustered by removing 57 edges (a number chosen originally because it gave a tractable number of clusters of a reasonable size in the Lehner dataset).

## Authors' contributions

RD: analysis, interpretation and writing. FD: statistical analysis. CS: data acquisition and supervision.

## Supplementary Material

Additional File 11 Additional file 11 shows the Lehner dataset before it was clustered. The Lsm proteins are highlighted. Click here for file

Additional File 12 Additional file 12 shows all the clusters produced when the Lehner dataset was clustered by removing 57 edges. The whole cluster containing the Lsm proteins is highlighted. Click here for file

Additional File 1 These files provide information on all the clusters (of size > 1) formed when the Gavin, Uetz and Lehner datasets were clustered. Each file name begins with the name of the dataset used in clustering, followed by the number of edges removed to perform the clustering. The files with a name ending 'clusters.txt' list all the members of each cluster and all the GO terms which were found to be significant for that cluster. Files ending with 'proteins-per-GO.txt', give further detail, showing the transcripts in the cluster which had the significant GO annotations. The examples provided show each dataset with the 'best' number of edges removed (see Results section: Difference in clustering between the datasets). For the smallest (Lehner) dataset, examples with greater and fewer numbers of edges removed are also provided. Click here for file

Additional File 2 These files provide information on all the clusters (of size > 1) formed when the Gavin, Uetz and Lehner datasets were clustered. Each file name begins with the name of the dataset used in clustering, followed by the number of edges removed to perform the clustering. The files with a name ending 'clusters.txt' list all the members of each cluster and all the GO terms which were found to be significant for that cluster. Files ending with 'proteins-per-GO.txt', give further detail, showing the transcripts in the cluster which had the significant GO annotations. The examples provided show each dataset with the 'best' number of edges removed (see Results section: Difference in clustering between the datasets). For the smallest (Lehner) dataset, examples with greater and fewer numbers of edges removed are also provided. Click here for file

Additional File 3 These files provide information on all the clusters (of size > 1) formed when the Gavin, Uetz and Lehner datasets were clustered. Each file name begins with the name of the dataset used in clustering, followed by the number of edges removed to perform the clustering. The files with a name ending 'clusters.txt' list all the members of each cluster and all the GO terms which were found to be significant for that cluster. Files ending with 'proteins-per-GO.txt', give further detail, showing the transcripts in the cluster which had the significant GO annotations. The examples provided show each dataset with the 'best' number of edges removed (see Results section: Difference in clustering between the datasets). For the smallest (Lehner) dataset, examples with greater and fewer numbers of edges removed are also provided. Click here for file

Additional File 4 These files provide information on all the clusters (of size > 1) formed when the Gavin, Uetz and Lehner datasets were clustered. Each file name begins with the name of the dataset used in clustering, followed by the number of edges removed to perform the clustering. The files with a name ending 'clusters.txt' list all the members of each cluster and all the GO terms which were found to be significant for that cluster. Files ending with 'proteins-per-GO.txt', give further detail, showing the transcripts in the cluster which had the significant GO annotations. The examples provided show each dataset with the 'best' number of edges removed (see Results section: Difference in clustering between the datasets). For the smallest (Lehner) dataset, examples with greater and fewer numbers of edges removed are also provided. Click here for file

Additional File 5 These files provide information on all the clusters (of size > 1) formed when the Gavin, Uetz and Lehner datasets were clustered. Each file name begins with the name of the dataset used in clustering, followed by the number of edges removed to perform the clustering. The files with a name ending 'clusters.txt' list all the members of each cluster and all the GO terms which were found to be significant for that cluster. Files ending with 'proteins-per-GO.txt', give further detail, showing the transcripts in the cluster which had the significant GO annotations. The examples provided show each dataset with the 'best' number of edges removed (see Results section: Difference in clustering between the datasets). For the smallest (Lehner) dataset, examples with greater and fewer numbers of edges removed are also provided. Click here for file

Additional File 6 These files provide information on all the clusters (of size > 1) formed when the Gavin, Uetz and Lehner datasets were clustered. Each file name begins with the name of the dataset used in clustering, followed by the number of edges removed to perform the clustering. The files with a name ending 'clusters.txt' list all the members of each cluster and all the GO terms which were found to be significant for that cluster. Files ending with 'proteins-per-GO.txt', give further detail, showing the transcripts in the cluster which had the significant GO annotations. The examples provided show each dataset with the 'best' number of edges removed (see Results section: Difference in clustering between the datasets). For the smallest (Lehner) dataset, examples with greater and fewer numbers of edges removed are also provided. Click here for file

Additional File 7 These files provide information on all the clusters (of size > 1) formed when the Gavin, Uetz and Lehner datasets were clustered. Each file name begins with the name of the dataset used in clustering, followed by the number of edges removed to perform the clustering. The files with a name ending 'clusters.txt' list all the members of each cluster and all the GO terms which were found to be significant for that cluster. Files ending with 'proteins-per-GO.txt', give further detail, showing the transcripts in the cluster which had the significant GO annotations. The examples provided show each dataset with the 'best' number of edges removed (see Results section: Difference in clustering between the datasets). For the smallest (Lehner) dataset, examples with greater and fewer numbers of edges removed are also provided. Click here for file

Additional File 8 These files provide information on all the clusters (of size > 1) formed when the Gavin, Uetz and Lehner datasets were clustered. Each file name begins with the name of the dataset used in clustering, followed by the number of edges removed to perform the clustering. The files with a name ending 'clusters.txt' list all the members of each cluster and all the GO terms which were found to be significant for that cluster. Files ending with 'proteins-per-GO.txt', give further detail, showing the transcripts in the cluster which had the significant GO annotations. The examples provided show each dataset with the 'best' number of edges removed (see Results section: Difference in clustering between the datasets). For the smallest (Lehner) dataset, examples with greater and fewer numbers of edges removed are also provided. Click here for file

Additional File 9 These files provide information on all the clusters (of size > 1) formed when the Gavin, Uetz and Lehner datasets were clustered. Each file name begins with the name of the dataset used in clustering, followed by the number of edges removed to perform the clustering. The files with a name ending 'clusters.txt' list all the members of each cluster and all the GO terms which were found to be significant for that cluster. Files ending with 'proteins-per-GO.txt', give further detail, showing the transcripts in the cluster which had the significant GO annotations. The examples provided show each dataset with the 'best' number of edges removed (see Results section: Difference in clustering between the datasets). For the smallest (Lehner) dataset, examples with greater and fewer numbers of edges removed are also provided. Click here for file

Additional File 10 These files provide information on all the clusters (of size > 1) formed when the Gavin, Uetz and Lehner datasets were clustered. Each file name begins with the name of the dataset used in clustering, followed by the number of edges removed to perform the clustering. The files with a name ending 'clusters.txt' list all the members of each cluster and all the GO terms which were found to be significant for that cluster. Files ending with 'proteins-per-GO.txt', give further detail, showing the transcripts in the cluster which had the significant GO annotations. The examples provided show each dataset with the 'best' number of edges removed (see Results section: Difference in clustering between the datasets). For the smallest (Lehner) dataset, examples with greater and fewer numbers of edges removed are also provided. Click here for file

Additional File 13 Additional file 13 shows more detail for this cluster, including the transcript ID for each node. The images were produced using the BioLayout [32] graph visualisation tool Click here for file

## Figures and Tables

**Table 1:** Datasets used for analysis Numbers of nodes and edges in each of the datasets used and a brief description of the methods used to generate the datasets.

Name	Nodes	Edges	Description and Reference
Gavin	1343	3145	Mass screen of yeast protein complexes using affinity purifica tion [15] (Note 1)
Ito	3271	4469	Mass screen of yeast protein interactions using Y2H [18] (Note1)
Lehner	329	406	Y2H interactions between *H. sapiens *proteins A dataset focused on RNA degradation and other MHCIII functions. [12, 13] (Note 2)
Uetz	1358	1498	Mass screen of yeast protein interactions using Y2H [14] (Note 1).

Notes: 1. The Gavin, Ito and Uetz graphs were all generated from BIND [28] derived datasets, which had GO annotations added and were supplied with v0.9.1 of the 'Osprey' graph visualisation tool [29,30]

2. The Lehner dataset is a combined set of the data from the two cited papers. These data are available in both IntAct [31] (experiment references EBI-348647, EBI-368082 and EBI-368083) and BIND [28] (refs 130691–130793 and 153087–153089)

**Table 2:** range of cluster sizes The distribution of cluster sizes in 3 datasets, after clustering with different numbers of edges removed.

Dataset	Number Edges Removed	Edges Re-moved %	Nodes per Cluster					
			
			1	2–5	6–20	21–50	51–200	201+
			
			Number of Clusters in Size Range					
Uetz	30	2%	13	128	9	3	0	1
Uetz	57	4%	13	128	9	3	1	1
Uetz	100	7%	13	128	11	5	4	1
Uetz	200	13%	13	130	32	19	1	0
Uetz	400	27%	21	256	71	0	0	0
								
Gavin	57	1.5%	0	33	8	2	0	1
Gavin	400	15%	0	33	16	4	3	2
Gavin	800	25%	4	58	57	15	2	0
Gavin	1500	50%	263	154	67	1	0	0
								
Lehner	15	4%	1	6	5	2	1	0
Lehner	30	7%	1	6	7	3	1	0
Lehner	57	14%	1	6	10	4	1	0
Lehner	100	25%	4	15	23	0	0	0

**Table 3:** cluster characteristics The average cluster size, number of clusters and other properties of the dataset, after clustering with different numbers of edges removed.

Dataset	Number of Edges Removed	Edges Removed %	Number of clusters size > 1	Average Cluster Size	Biggest cluster(%)	Single Nodes(%)
Uetz	30	2%	141	9.5	849(61%)	13(1 %)
Uetz	57	4%	142	9.5	715(53%)	13(1 %)
Uetz	100	7%	149	9.0	459(38%)	13(1 %)
Uetz	200	13%	182	7.4	53(4 %)	13(1 %)
Uetz	400	27%	327	4.1	13(1 %)	21(1.5%)
						
Gavin	57	1.5%	44	30.5	1106(82%)	0(0 %)
Gavin	400	15%	58	23.1	360(27%)	0(0 %)
Gavin	800	25%	132	10.1	56(4 %)	4(0.3%)
Gavin	1500	50%	222	4.9	23(2 %)	263(19 %)
						
Lehner	15	4%	14	23.4	190(58%)	1(0.3%)
Lehner	30	7%	17	19.3	143(43%)	1(0.3%)
Lehner	57	14%	21	15.6	60(18%)	1(0.3%)
Lehner	100	25%	38	8.6	19(6 %)	2(0.6%)

**Table 4:** cluster quality Association between the size of the clusters and the quality and quantity of significant GO terms with different numbers of edges removed.

Dataset	Number of Edges Removed	Edges Removed %	GO per Cluster	GO per Node	Depth of GO per Node	Number of Clusters with no significant annotation
Uetz	30	2%	0.7	0.1	4.9	120
Uetz	57	4%	0.8	0.1	4.9	120
Uetz	100	7%	0.9	0.1	4.9	121
Uetz	200	13%	1.1	0.2	4.8	137
Uetz	400	27%	3.5	0.7	4.5	261
						
Gavin	57	1.5%	2.1	0.1	4.6	23
Gavin	400	15%	3.4	0.2	4.7	24
Gavin	800	25%	2.8	0.3	4.6	59
Gavin	1500	50%	2.2	0.5	4.6	336
						
Lehner	15	4%	22.9	1.0	5.8	1
Lehner	30	7%	21.3	1.1	5.8	1
Lehner	57	14%	19.2	1.2	5.8	1
Lehner	100	25%	15.5	1.8	5.8	2

**Table 5:** significant GO terms for the Lehner dataset A selection of GO terms with significant correlations to the 20 clusters in the Lehner dataset, clustered by removing 57 edges. (The numbers after the descriptions show the proportion of proteins in the cluster which were annotated with that GO term). The complete set of GO terms for each of these clusters can be seen in Additional file 7 and the identity of the transcripts associated with the significant GO terms can be found in Additional file 8.

Cluster Number	Size of Cluster	Significant GO descriptions
15	20	ubiquitination 4/20
4	49	protein biosynthesis 7/49, RNA catabolism 4/49, translation 3/49
19	3	ubiquitin 1/3, cell defence 1/3
8	24	electron transport 2/24
11	10	transcription regulation3/10
16	22	transport 6/22, glucose catabolism 2/22
18	7	DNA repair 2/7
3	60	RNA splicing 14/60, spliceosome 5/60
7	10	ribosome assembly 2/10, cytoplasmic exosome 1/10
12	8	protein metabolism 3/8, phosphorylation 3/8
22	1	morphogenesis 1/2, membrane 1/2
2	19	signal transduction 4/19, ER 3/19
9	4	transcription reg 1/4
21	4	mRNA catabolism 1/4
6	12	mRNA export 1/12, DNA binding 4/12
1	18	cytoskeleton 3/18
20	2	ATP biosynthesis 2/2
14	14	DNA replication 2/14, cell cycle 3/14
10	23	biological process 8/23 oncogenesis 2/23

**Table 6:** the distribution of proteins associated with RNA metabolism from TAP-C128 The number of proteins from TAP-C128 [15] which cluster together when different numbers of edges are removed and also the proportions which are annotated for RNA metabolism.

Edges removed	Number of clusters associated with the GO term 'RNA metabolism'	Largest group of TAP-C128 found together	Proportion of proteins *	Number of Lsm proteins in this cluster
57	3 clusters	36/36	128/1106	7/7
400	7 clusters	27/36	57/142	7/7
800	8 clusters	13/36	43/56	7/7
1500	11 clusters	7/36	5/7	6/7

*Proportion of proteins in the cluster containing most TAP-C128 proteins which were associated with RNA metabolism

**Table 7:** the distribution of affinity purified proteins from TAP C-162 TAP C-162 [15] is an mRNA polyadenylation complex of 36 proteins, thought to be a stable complex

Edges Removed	Number of Clusters Containing TAP C-162 proteins	Numbers of the TAP C-162 proteins in each of the Clusters
57	1	(36)
400	5	(25,7, 4 × 1)
800	9	(23,4, 9 × 1)
1500	16	(16,22, 16 × 1)

**Table 8:** the distribution of affinity purified proteins from TAP C-151 TAP C-151 [15] is a signaling protein complex of 45 proteins, thought to be more labile than TAP C-162

Edges Removed	Number of Clusters Containing TAP C-151 proteins	Numbers of the TAP C-151 proteins in each of the Clusters
57	1	(45)
400	3	(43,1,1)
800	11	(14,12,7,3,2,2, 5 × 1)
1500	21	(9,7,6,4,3,2, 14 × 1)

**Table 9:** datasets used to investigate false positives These datasets were used to investigate the effect of false positive edges on the clustering of the datasets

Name	Nodes	Edges
Lehner original	329	406
Lehner plus False Positive proteins and edges	353	465
Lehner with false positive proteins' edges disconnected	353	397
Ito [18]	3271	4469

**Table 10:** cluster size distribution with and without false positives

Dataset	Number Edges Removed	Edges Removed %	Nodes per Cluster					
			
			1	2–5	6–20	21–50	51–200	201+
			
			Number of Clusters in Size Range					
Lehner	57	14%	1	6	10	4	1	0
Lehner plus False Positive Edges(FPE)	57	12.3%	1	6	11	2	2	0
Lehner minus 68 FPE	57+68*	26.9%	32	6	9	5	1	0
Lehner random edges removed**	57+68*	26.9%	39.7 ± 3.2	6.5 ± 1.0	13.4 ± 2.1	4.1 ± 1.1	0.05 ± 0.2	0 ± 0
Ito minus 26 FPE	57+26*	1.9%	25	183	4	0	0	1
Ito minus 26 random edges	57+26*	1.9%	11	189	5	0	0	1

* edges removed for clustering + false positive or random edges removed

**Lehner plus FPE with 68 edges removed at random (for 100 replicates mean ± standard deviation)

**Table 11:** cluster characteristics with and without false positives

Dataset	Number of Edges Removed	Edges Removed %	Number of clusters size > 1	Average Cluster Size	Biggest cluster(%)	Single Nodes(%)
Lehner	57	14%	21	15.6	60(18 %)	1(0.3 %)
Lehner plus False Positive Edges (FPE)	57	12.3%	21	16.8	67(19.0%)	1(0.3 %)
Lehner (FPE edges removed)	57+68*	26.9%	21	15.3	56(15.9%)	32(9.0 %)
Lehner random edges removed**	57+68*	26.9%	24.0 ± 1.5	13.1 ± 0.8	39.2 ± 6.1(11.1%)	39.2 ± 3.2(11.2%)
Ito minus FPE	57+26*	1.9%	188	17.3	2798(85.5%)	25(0.8 %)
Ito minus 26 random edges	57+26*	1.9%	195	16.7	2787(85.2%)	11(3.4 %)

* edges removed for clustering + false positive or random edges removed

**Lehner plus FPE with 68 edges removed at random (for 100 replicates mean ± standard deviation)

**Table 12:** cluster quality with and without false positives

Dataset	Number of Edges Removed	Edges Removed %	GO per Cluster	GO per Node	Depth of GO per Node	Number of Clusters with no significant annotation
Lehner	57	14%	19.2	1.2	5.8	1
Lehner plus False Positive Edges (FPE)	57	12.2%	18.2	1.1	5.7	1
Lehner (FPE edges removed)	57+68*	26.9%	19.33	1.3	5.7	10
Lehner random edges removed**	57+68*	26.9%	17.5 ± 4.6	1.3 ± 0.4	5.7 ± 0.08	4.2 ± 5.2
Ito minus FPE	57+26*	1.9%	1.3	0.1	4.7	149
Ito minus 26 random edges	57+26*	1.9%	1.3	0.1	4.7	146

* edges removed for clustering + false positive or random edges removed

**Lehner plus FPE with 68 edges removed at random (for 100 replicates mean ± standard deviation)

**Table 13:** Clustering of RNA splicing proteins in the Lehner dataset with different numbers of edges removed.

edges removed	size of 'RNA splicing' cluster	proportion of proteins annotated for 'RNA splicing'	proportion of proteins which were prey of Lsm proteins in [13]
15	190	18/190	51/190
30	143	17/143	49/143
57	60	14/60	49/60
100	17	10/17	14/17
