# Supplementary figures and images for: The Use of Edge-Betweenness Clustering to Investigate Biological Function in Protein Interaction Networks
Source: BMC Bioinformatics. 2005 Mar 1;6:39. doi: 10.1186/1471-2105-6-39 (PMC555937; doi:10.1186/1471-2105-6-39)

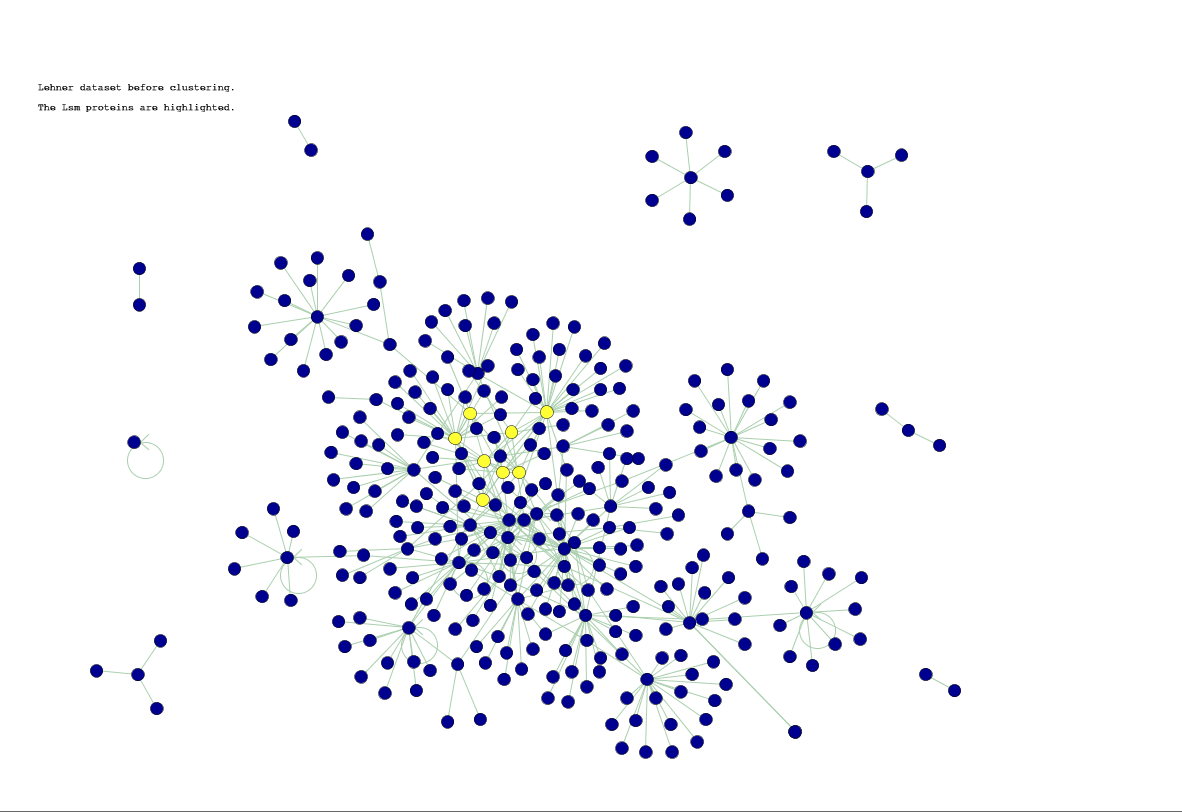

Supplement: Additional File 11 — shows the Lehner dataset before it was clustered. The Lsm proteins are highlighted. [file 1471-2105-6-39-S11.png]

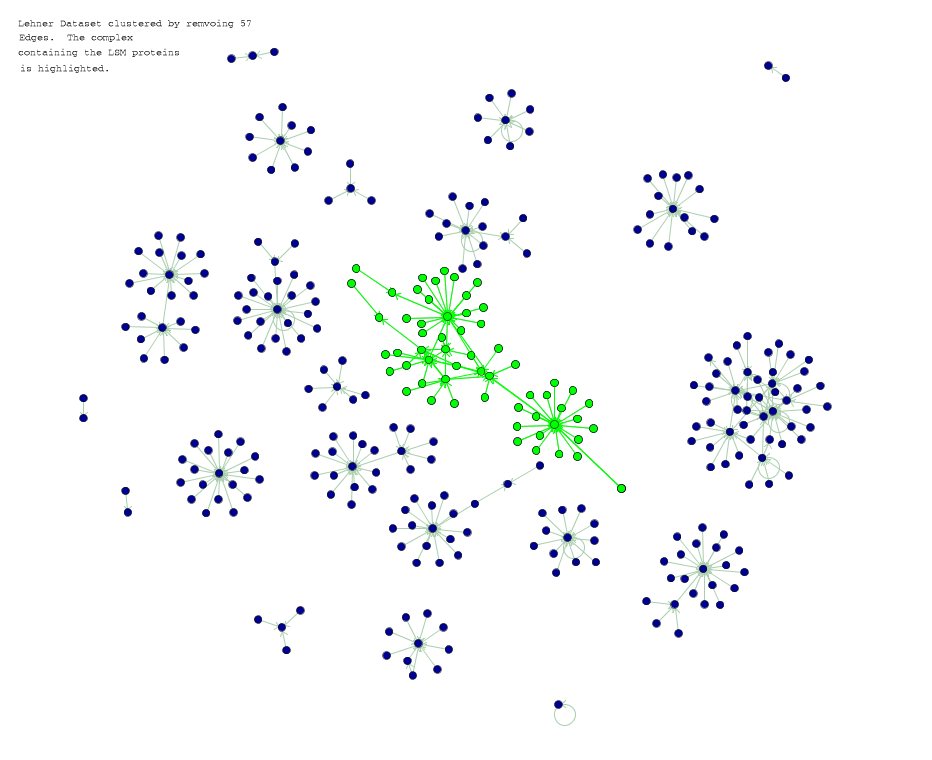

Supplement: Additional File 12 — shows all the clusters produced when the Lehner dataset was clustered by removing 57 edges. The whole cluster containing the Lsm proteins is highlighted. [file 1471-2105-6-39-S12.png]

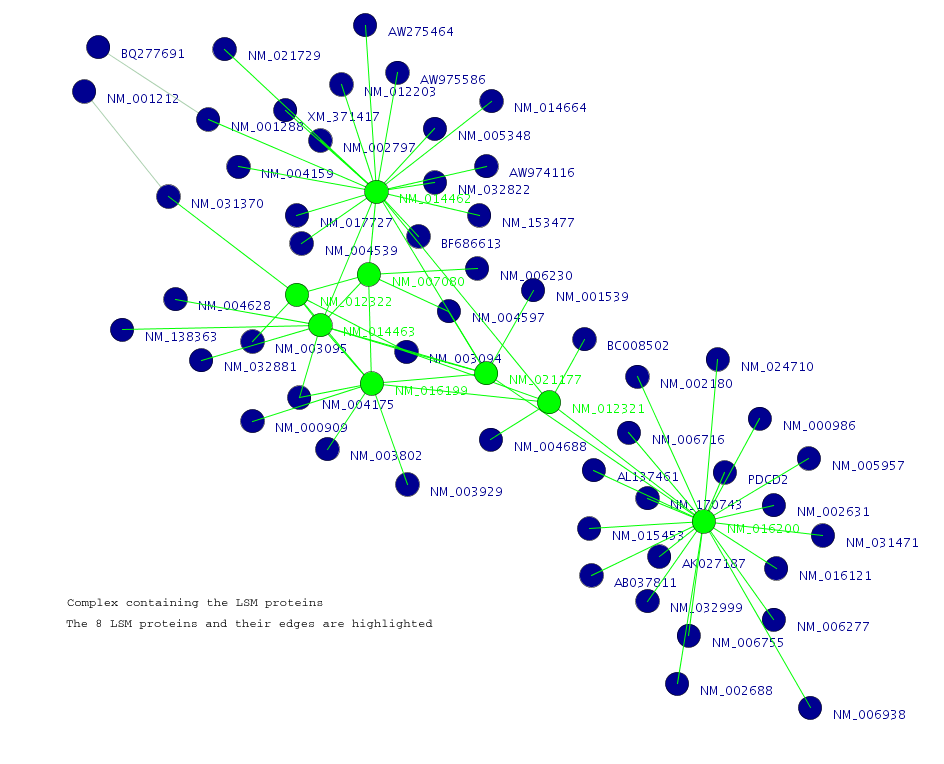

Supplement: Additional File 13 — shows more detail for this cluster, including the transcript ID for each node. The images were produced using the BioLayout [32] graph visualisation tool [file 1471-2105-6-39-S13.png]
